# Accurate Monte Carlo simulation of frequency‐domain optical coherence tomography

**DOI:** 10.1002/cnm.3177

**Published:** 2019-03-07

**Authors:** Yan Wang, Li Bai

**Affiliations:** ^1^ School of Computer Science University of Nottingham Nottingham NG8 1BB UK

**Keywords:** Monte Carlo simulation, optical coherent tomography

## Abstract

Optical coherence tomography (OCT) relies on optical interferometry to provide noninvasive imaging of living tissues. In addition to its 3D imaging capacity for medical diagnosis, its potential use for recovering optical parameters of biological tissues for biological and pathological analyses has also been explored by researchers, as pathological changes in tissue alter the microstructure of the tissue and therefore its optical properties. We aim to develop a new approach to OCT data analysis by estimating optical properties of tissues from OCT scans, which are invisible in the scans. This is an inverse problem. Solving an inverse problem involves a forward modeling step to simulate OCT scans of the tissues with hypothesized optical parameter values and an inverse step to estimate the real optical par1meters values by matching the simulated scans to real scans. In this paper, we present a Monte Carlo (MC)–based approach for simulating the frequency‐domain OCT. We incorporated a focusing Gaussian light beam rather than an infinitesimally thin light beam for accurate simulations. A new and more accurate photon detection scheme is also implemented. We compare our MC model to an analytical OCT model based on the extended Huygens‐Fresnel principle (EHF) to demonstrate the consistency between the two models. We show that the two models are in good agreement for tissues with high scattering and high anisotropy factors.

## 1 INTRODUCTION

**Figure 1:**
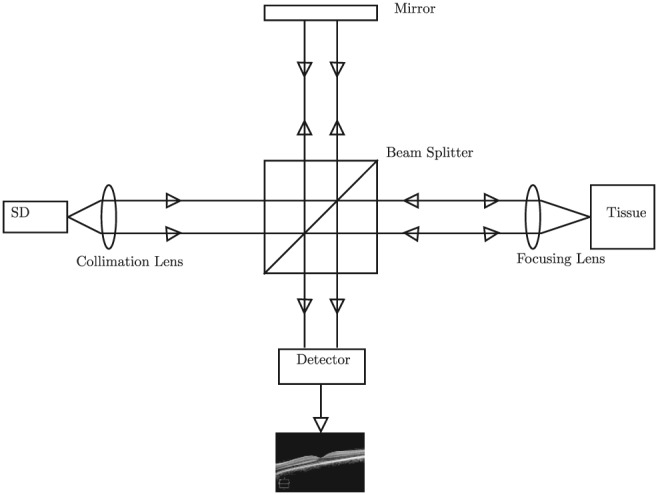
A diagram of a generic OCT system. SD, super‐luminescent diode

In order to simulate OCT, we must have some knowledge how OCT works. The fundamental theory behind OCT is ranging measurement using low‐coherence interferometry in physics. A simplified diagram of an OCT system is shown in Figure 1. The OCT system emits a light beam from its light source, which is then split into two arms by a splitter: a reference arm and a sample arm. The light through the reference arm is cast onto a mirror and reflected back, thus experiencing a time delay. The light exiting the sample arm is guided through a fiber and focused onto the sample tissue to be examined. The backscattered or reflected light from the tissue is redirected back to the system through the same fiber and combined with the returning light through the reference arm. The mixed light creates an interference pattern on the surface of a photoreceiver or detector, which is then converted into electronic signals. The signals are further processed into a reflectivity profile of the sample tissue at various scanned depth, which is called the A‐scan. As the sample arm sweeps laterally across the tissue sample, many A‐scans are generated along the lateral direction and can be combined into a cross‐sectional 2D image, namely, the B‐scan image. B‐scans resulted from different lateral directions can be further combined into a 3D volume dataset.

There are mainly two types of OCT systems widely used today: time‐domain OCT (TDOCT) and frequency‐domain OCT (FDOCT).^1^ FDOCT includes spectral‐domain (or spectrometer‐based) OCT (SDOCT) and swept‐source OCT (SSOCT). In TDOCT, the path length of the reference arm light is changed during the acquisition of an A‐scan. The reference beam light interferes with the sample arm light scattered from a scattering site at a particular depth of the tissue of which the path length falls within the coherence length. The path length of the reference arm is then accommodated continuously to construct the whole A‐scan. A disadvantage of TDOCT is that the scanning process is slow. Any relative movement between the OCT system and the examined medium would lead to artifacts in the resulting signal. FDOCT features a light source of a wide wavelength bandwidth, and the interfered light between the sample and reference arms is dispersed by a spectrometer, resulting in a spectrum. An inverse Fourier‐transform is then applied to convert the spectrum into a depth scan. Unlike TDOCT, only one scan is needed to acquire a full depth scan. The SSOCT, on the other hand, utilizes a light source of a single frequency. During a scan, the light source quickly sweeps a series of frequencies, and the interference pattern from each frequency is recorded continuously by a photo‐receiver. The A‐scan may be constructed similar to FDOCT via an inverse Fourier transform.

In this paper, we first review briefly some related work. In Section 3, we present the details of our MC approach for simulating frequency‐domain OCT. Unlike conventional MC approaches for OCT simulation, we use focusing Gaussian light beams instead of infinitesimally thin beams as the input light source and incorporate a new photon detection scheme for the focusing Gaussian light beam. We then compare our simulation results with the analytical EHF model in Section 4, followed by a discussion of some of the related issues.

## 2 RELATED WORK

There are generally two major approaches for modeling the imaging process of OCT. One uses an analytical model of the OCT derived from the theories of optics, such as the extended Huygens‐Fresnel principle (EHF) and the radiative transfer equation (RTE) under the small angle approximation. Yi et al^2^ proposed an analytical model termed ISOCT for analysing frequency‐domain OCT signals. The model assumes that the medium has continuous refractive index (RI) fluctuation, which is specified as an RI correlation function using the Whittle‐Matern functional family. By deducing the RI correlation function from OCT signals, one is able to estimate a full set of optical properties of the tissue. It is a single scattering model, which only takes into account photons scattered once before it is back‐propagated into the detector. In reality, however, multiple scattered light may contribute to the OCT signals as well. As photons are transmitted to a certain depth through tissues with random spatial variation in the refraction index (ie, inhomogeneous), a large number of them are highly likely to be scattered for multiple times. Ignoring these multiple scattering events may introduce errors in the estimation of optical parameters. To account for multiple scattering events, Thrane^3^ applied the EHF principle to solve the Maxwell equations for light wave propagation through tissues. Optical parameters of tissues such as the scattering coefficient and the effective anisotropy factor are estimated by fitting the model to real OCT A‐scans. This model is flexible in the sense that it can be applied to a variety of OCT geometry by incorporating ABCD matrices into their EHF framework. An ABCD matrix can be used to trace light rays through an optical system. Turchin et al^4^ proposed an analytical OCT model based on the RTE to solve the inverse problem of OCT. The RTE cannot be directly applied for OCT modeling since it does not take into account the coherence between the reference and the sample arms, which is essential for OCT imaging. Therefore, their model assumes a delta‐correlated light source and has accounted for the correlation between light propagated forward and backward. Interestingly, it was confirmed in Drexler and Fujimoto^5^ that the RTE model with the small angle approximation and the EHF model are equivalent, if both models adopt the same scattering phase function.

The other major approach is based on MC simulation. This approach simulates directly the interaction between photons and tissues during light transport within the tissue.^6^, ^7^, ^8^ Given the optical geometry and optical properties of a tissue model, photons are simulated and tracked along their path through the tissue subject to stochastic events of absorption and scattering. Wang et al^9^ developed a simulator, namely, the MCML, for studying the propagation of a laser beam incident to multilayered tissues. The simulator however can only deal with homogeneous and isotropic media. Other simulators have been developed based on MCML for TDOCT.^10^, ^11^, ^12^, ^13^ Previous work implemented the feature of low‐coherent interference of sample and reference arms by taking into account the coherence length of the light source in detecting photons. They differ mainly in how the geometry of scattering media was structured, or whether an importance sampling scheme was incorporated to boost the simulation performance. As OCT image formation relies on back‐scattered photons, photon packets reflected from the tissue are detected subject to a photon detection scheme. Back‐scattered photons are differentiated as class I photons and class II photons.^14^ Class I photons are largely single‐scattered photons from the ballistic component of the sample arm, which have reached a scan depth z and of which the optical path length differs from that of the reference arm no larger than the coherence length of the optical source. Class II photons are the same as class I photons except that they have not reached the scanned depth but has a comparable path length to the length of the reference arm due to multiple scattering, which mainly contribute to diffusive reflectance. The backscattered reflectance at depth z contributed by class I photons, and the diffusive reflectance contributed by class II photons are estimated by summarizing the weights of all II photons. In practice, the whole scan depth needs to be discretized into a grid with a resolution limited by the coherent length.

MC‐based approaches can give very realistic simulation results containing speckle noises, which are inherent in real OCT images and are applicable for tissues with complex geometry or optical properties. However, a major drawback of the approach is that photons are treated as physical particles, and the general MC approaches are incapable of modeling wave phenomena such as diffraction.^15^ Another drawback is that the computation for solving the inverse problem can be very costly, since a large quantity of photon packets have to be traced in simulation in order to obtain a sufficient number of photons back scattered from the photon‐tissue interaction for every depth of the complete scan range. To tackle the inefficiency problem, importance sampling has been applied to the photon scattering step to greatly enhance the chance for photon packets being back‐scattered towards the photon detector.^12^, ^16^

Some MC simulation studies focused on simulating FDOCT.^17^, ^18^ In the FDOCT simulation, the light source is modeled as one with a broad wavelength bandwidth. The photon propagation is similar to TDOCT except that FDOCT simulation does not differentiate class I and II photons. The difference is largely at the detector side. The photon detector is modeled as a wavenumber‐resolved photo‐detector array, and each detector corresponds to a certain wavenumber. Once a photon has been detected, each detector will deposit its weight modulated by a wave function of the detector's wavenumber and the photon's optical path length. Hence, a wavenumber‐dependent fringe signal encoded with optical path lengths is obtained after simulation, followed by an inverse Fourier transform to reconstruct the A‐scan. FDOCT simulation can alleviate the inefficiency problem mentioned above, since each detected photon contributes to the spectrum across all wavenumbers, which in turn helps reconstruction of A‐scans for the full depth range. We are also aware of a similar photon propagation procedure used in a more recent work^19^ for simulating optical tomography (OT) in the frequency domain. The weight of each photon was also modulated by a complex wave form as a function of the optical path length. However, because the principle of OT imaging is different from OCT, the photon detection and formation of scans in the OT simulation are very different from the OCT simulation.

## 3 MONTE CARLO SIMULATION OF OCT

### 3.1 A framework for simulating photon propagation in tissues

**Figure 2:**
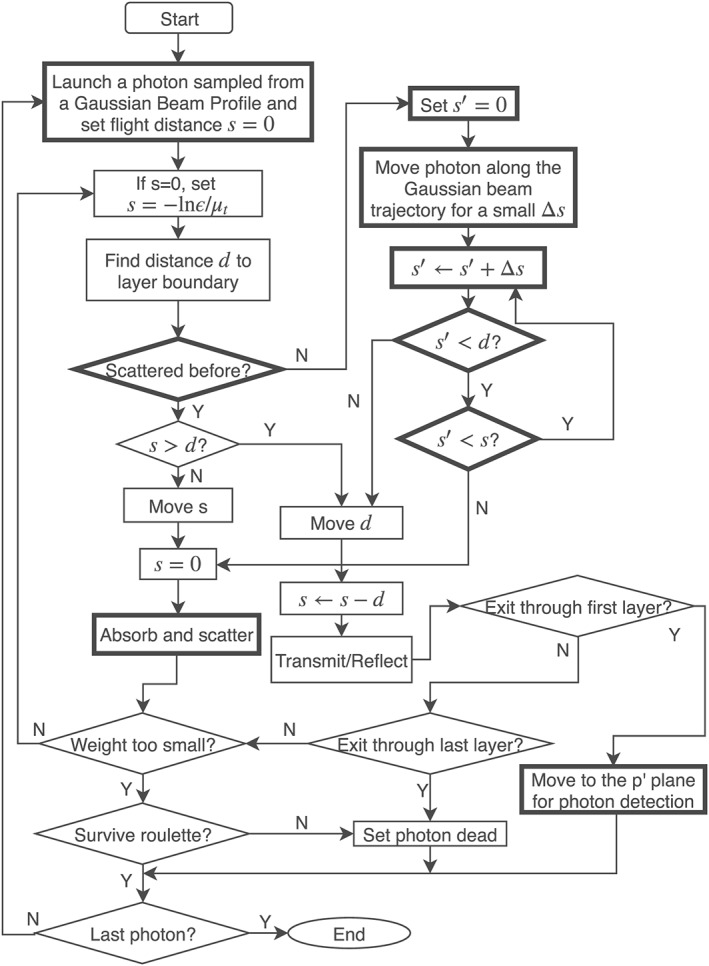
A flow‐chart of our simulation framework

**Figure 3:**
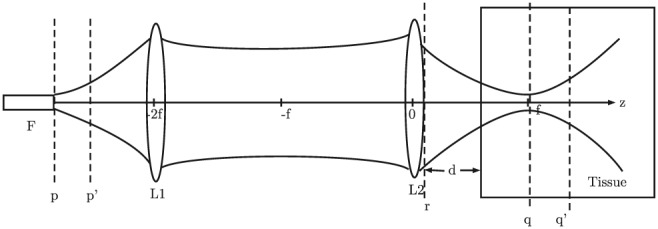
A 4F optic system

A flow‐chart of the MC simulation framework is shown in Figure 2 adapted from the original MCML framework.^9^ The modified components of the original MCML are in represented with frames of thicker boundaries than the rest in the flow‐chart. The OCT system geometry adopted in our approach is shown in Figure 3. For simplicity, only the sample and reference arms are presented. The sample arm is equipped with a 4F optic system, composed of an emitting optical fiber F, a collimating lens L1, and a focusing lens L2. L1 and L2 have the same focal length *f*. F is placed at a focal point on one side of L1. L2 is at a distance 2F away from L1 on the other side, and *d* is distance away from the tissue. L1 and L2 share a common focal plane located between them. The sample light beam emitted from F is casted onto L1 and becomes collimated. It then goes through L2 and is focused into a tissue. F is situated at the focal plane of L1 (p‐plane). The light beam is focused at the focal plane of L2 (q‐plane). A reference light beam goes through another 4F system and is reflected back by a mirror situated at the arm end. The sample light beam has a 1/*e* radius equal to *w*_*f*_ at the fiber end and *w*_0_ on the r‐plane. For the convenience of analysis, it can be regarded that the backscattered sample light is mixed with the reference light beam at the r‐plane, which is equivalent to the mixing of both at the photodetector.^3^ The p‐plane and q‐plane are a pair of conjugate planes of the 4F system.

Tissues are modeled as multilayered with constant thickness each layer. The coordinate origin is at the top surface of the tissue, and the z axis is perpendicular to all layers and the x and y axes are parallel to the layers. Photons are modeled as photon packets with an initial weight set to unity. Photon packets are sampled from a Gaussian distribution of the intensity of a focusing light beam launched from the surface of a tissue model. Each photon packet is propagated through the tissue following a focusing Gaussian beam model before it encounters the first scattering event. The distance of its free movement is sampled from an exponential distribution specified with the absorption coefficient μ_a_ and the scattering coefficient μ_s_. After the free movement, the weight of the photon packet is reduced by the amount μ_a_ w/(μ_a_ + μ_s_). The scattering step updates the photon's movement direction by sampling a direction from a scattering phase function p(θ). The phase function is for modeling the distribution of photons being scattered along a direction specified by the scattering angle θ relative to its incident direction. In a cylindrical coordinate system with the z‐axis along the incident direction:

(1) $$\frac{1}{4\pi }\int_{0}^{2\pi }\int_{0}^{\pi }p(\theta )\sin\theta d\theta d\phi =1,$$

where ϕ is the azimuthal angle and p(θ) is normalized so that its integral over the full solid angle is one. The well‐known Henyey‐Greenstein (HG) phase function^20^ is often used for modeling scattering in biological tissues. However, in order to compare the MC simulation results with those of the EHF model, we replace the HG function with the small‐angle phase function.^3^, ^4^ It consists of two parts: a Gaussian density and an isotropic density, which is defined as

(2) $$x(z,\theta )=(1-2p_{b}(z))x_{1}(z,\theta )+2p_{b}(z).$$

Here, p_b_ is the backscattering probability of photons. x_1_(z,θ) is defined as a Gaussian density function modeling forward scattering at depth z

(3) $$x_{1}(z,\theta )=\frac{2}{1-g(z)}\exp\left(-\frac{1-\cos\theta }{1-g}\right)\approx \frac{4}{\left\langle \theta^{2}(z)\right\rangle }\exp\left(-\frac{\theta^{2}}{\left\langle \theta^{2}(z)\right\rangle }\right),$$

where ⟨θ ^2^(z)⟩ is the variance of the scattering angle at depth z, which can be approximated as a function of the anisotropic factor g(z) at depth z when the scattering angle is small: ⟨θ ^2^(z)⟩≈2(1 − g(z)). To sample a scattering angle is simple, we firstly generate a uniform random number ϵ. If ϵ is smaller than 2p_b_, we sample the scattering angle uniformly from the range [0,π]. Otherwise, we sample from the Gaussian density function.

Once the photon packet hits a boundary of the tissue, the Fresnel equations are used to determine whether it should be transmitted to the next layer or be reflected at the boundary. If the photon packet exits from the tissue, it will be terminated and is subject to a photon detection scheme to determine if it contributes to the formation of OCT signals. If the weight of the photon packet drops below a threshold, it is then terminated subject to a Russian survive roulette. The procedure is repeated for many times to simulate a large number of photons. Finally, the spectrum data obtained from the simulation is converted to OCT A‐scans using the inverse Fourier transform.

### 3.2 Simulating FDOCT

The implementation of FDOCT simulation was based on the method of Hartinger et al,^18^ which is briefly presented here. Let N be the number of frequency components, δk the wavenumber linear spacing, and k_0_ be the smallest wavenumber. The wavenumber span of the light source is then (k_0_,k_0_ + δk,k_0_ + 2δk,…,k_0_ + (N − 1)δk). Let us assume a photon emit from a wave component having wavenumber k hits a reflector at depth z_0_ in the tissue and is backscattered towards the detector. Its optical path length is then 2z_0_, and it creates an electric field at the detector, which can be expressed as

(4) $$E_{i}=\sqrt{W_{i}}\exp(2iz_{0}k),$$

where W_i_ is the weight of the ith photon packet. In essence, this wave form of the photon has encoded the depth z_0_, which can be reconstructed by taking the Fourier transform of equation (4), regarding k as time and z_0_ as frequency. By collecting contributions from all detected photons, we obtain a spectrum‐like signal as a function of the wavenumber^18^:

(5) $$S(k)=\sqrt{G(k)}\sum_{i=1}^{N}\sqrt{W_{i}}\exp(2ikz_{i}),$$

where G(k) is the light source spectrum and N is the total number of detected photon packets. Assuming a Gaussian spectrum, it can be defined as

(6) $$G(k)\propto \exp\left[-{\left(\frac{k-k_{c}}{\Delta k}\right)}^{2}\right],$$

where k_c_ is the central wavenumber and Δk is the wavenumber bandwidth. In case of SSOCT, G(k) can be simply set to unity. Generally, we treat the reference electric field as $R(k)=\sqrt{\alpha G(k)}$, where α is a parameter chosen to scale the total sample arm power |S(k)|^2^ between 0.001% and 0.01% of the reference arm power |R(k)|^2^. Therefore, a spectrum as a function of the wavenumber is given by interfering the sample and reference arm fields^18^ as

(7) $$I_{D}(k)=|S(k)+R(k){|}^{2}-|S(k)-R(k){|}^{2}.$$

We further apply a Hamming window and the inverse Fourier Transform to the spectrum to recover the A‐scan. One convenient aspect of the FDOCT simulation is that it can be easily incorporated into the existing TDOCT simulation framework. Only the steps of photon collection and signal formation need to be modified. In Zhao^17^ and Hartinger et al,^18^ the magnitude of the inverse Fourier transform of I_D_(k) was taken as the A‐scan. We found that this does not give a consistent result as TDOCT under the same simulation configuration. We use the square of the magnitude instead. Finally, the imaging depth is calculated according to the Nyqist‐Shannon sampling theorem as

(8) $$D=\frac{1}{4}\frac{\lambda_{c}^{2}}{\delta \lambda },$$

where λ_c_ is the central wavelength λ_c_ = 2π/k_c_ and δλ is the wavelength spacing of the light source spectrum. In reality, FDOCT signals are obtained from a spectrometer as a spectrum specified upon a wavelength array. In the specification of OCT equipment, the minimum wavelength λ_min_, the central wavelength λ_c_, the wavelength bandwidth Δλ, and wavelength spacing δλ are often supplied. Before simulation, we then have to convert the specification into the wavenumber domain. Firstly, we need to resample the wavelength array by interpolation so that the corresponding wavenumbers will be evenly spaced after conversion. An interpolating factor s_i_ is calculated as^21^

(9) $$s_{i}={\left[\frac{1}{\lambda_{max}}+\frac{i}{M-1}\left(\frac{1}{\lambda_{min}}-\frac{1}{\lambda_{max}}\right)\right]}^{-1}-\lambda_{min},$$

where M is the length of the wavelength array and λ_max_ = λ_min_ + (M − 1)δλ. Then a new array of wavelength is obtained as λ_i_ = λ_min_ + s_i_. Thus, the wavenumber of each element of the interpolated new array is k_i_ = 2π/λ_i_. Finally, according to Izatt and Choma,^1^, eq2.8 the wavenumber bandwidth is calculated as

(10) $$\Delta k=\frac{\pi }{\sqrt{\ln2}}\frac{\Delta \lambda }{\lambda_{c}^{2}}.$$

### 3.3 Simulating focusing Gaussian beam

In OCT modeling, an important factor to consider is the incident light beam. A common practice in MC approaches is to model it as a photon stream with an infinitesimally small radius, but this is not true in reality. In analytical models such as EHF and RTE, the incident light is modeled as a focusing Gaussian beam and a simple focusing beam geometries can be incorporated. Due to diffraction of light waves, a Gaussian beam focuses to a spot with a diffraction‐limited size, which is also termed the beam wrist. However, conventional MC simulation approaches such as MCML do not take into account wave characteristics of photons since they are treated as particles. Nonetheless, a simple method is to use focusing geometry and direct photons towards the focal point before they are scattered, but the ballistic photons reaching the focal point will concentrate into a infinitesimally small point instead of forming the beam wrist. In our simulation, we adopt an alternative method for simulating focusing geometries proposed in Hokr et al.^15^ Photons are initially sampled from a Gaussian distribution of the intensity of an incident beam at the lens plane. Before encountering the first scattering event, each photon follows a trajectory where its moving direction is always perpendicular to the propagating wave front of the focusing Gaussian beam. As a result, the distribution of ballistic photons at any plane transversal to the optical axis is always Gaussian. After being scattered, a photon will propagate in a way as specified in traditional MC simulation. A great advantage of this method is that it can be easily integrated into existing conventional simulation frameworks such as MCML. Here, unlike Hokr et al,^15^ which assumes a focusing lens at z = 0 and presents a focusing Gaussian beam model in a tissue with the refractive index set to unity, we consider a more general setup where the focusing lens is positioned at d distance away from the surface of the medium and the refractive index of the medium is n.

Let w_0_ and w_f_ be the 1/e radius of the beam intensity at the focusing lens and the focal plane, respectively. In a medium with a refractive index n, w_f_ can be calculated as w_f_ = λf/(2nπw_0_), where λ is the wavelength of the focusing Gaussian beam and f the focal length. The power of the beam is given as $P=I_{0}\pi w_{f}^{2}$, where I_0_ is the optical intensity at the center of the beam wrist. The intensity profile of the beam at depth z is expressed as

(11) $$I(r;z)=I_{0}\frac{w_{f}^{2}}{w{\left(z\right)}^{2}}\exp\left[-\frac{r^{2}}{w{\left(z\right)}^{2}}\right],$$

where **r** is a position on the transverse plane. w(z) is the radius of the beam at depth z. Rather than sampling photons from the lens plane, we directly launch photons on the surface of the medium. There are two reasons for this. Firstly, there is no need to propagate photons from the focusing lens to the surface. Secondly, if there is mismatch in refractive indexes between the tissue and the air, a lot of photons launched from the lens may be directly reflected on the tissue surface. These photons are wasted since they have never entered the tissue. Hence, we can improve the simulation efficiency by directly sampling photons at the tissue surface to avoid possible reflection. To this end, we obtain the intensity distribution of the beam cast on the surface using Equation 11 with the radius w_s_ calculated as

(12) $$w_{s}\equiv w(z=0)=w_{f}\sqrt{1+{\left[\frac{\lambda (f-d)}{2\pi w_{f}}\right]}^{2}}.$$

After sampling a photon packet from this distribution, it is propagated into the medium using the same photon propagation procedure described in Hokr et al.^15^ The radius of the wave front at a depth z in the medium is given as

(13) $$R(z)=-(d-f+z/n)\left[1+{\left(\frac{z_{R}}{d-f+z/n}\right)}^{2}\right],$$

where z_R_ is the Rayleigh length calculated as $z_{R}=2n\pi w_{f}^{\prime 2}/\lambda$. $w_{f}^{\prime }$ is the radius of the beam at the focal plane in the medium: $w_{f}^{\prime }=\lambda (f-d)/(2n\pi w_{s})$. Assume at a time step t, the photon is at a location (x(t),y(t),z(t))^T^. Its direction of travel at t is defined as

(14) $$v(t)=\frac{1}{\sqrt{1+(x{\left(t\right)}^{2}+y{\left(t\right)}^{2})/R{\left(z(t)\right)}^{2}}}{\left(-\frac{x(t)}{R(z(t))},-\frac{y(t)}{R(z(t))},1\right)}^{T},$$

which is normal to the wave front of the Gaussian beam passing the photon's location. Given a small time step of propagation, the position **r**(t + 1) of the photon at the time step t + 1 is updated according to the equation of motion $\dot{r}(t)=(c/n)v(t)$ where c is the light speed. The equation can be solved to obtain the position of the photon at t + 1 by the Runge‐Kutta method using the second derivative of Equation 14.

### 3.4 The photon detection scheme

Once a photon packet leaves the tissue through the top surface, it may be detected by a photodetector. Some criteria must be satisfied in order for a photon packet to contribute to the OCT signal, which are referred to as a detection scheme.^22^ Previously published papers specified acceptance criteria on the angle and position of exiting photon packets related to the numerical aperture of the receiving lens or optical fibers in addition to the condition that the difference between the optical path length of the photon packet and the reference arm length must be less than the coherence length. Specifically, the angle between the incoming direction of the photon packet and the optical axis must be smaller than an acceptance angle of the receiving fiber or lens according to considerations of field distributions. However, Tycho^22^ argued that such considerations may not be valid since simulated photon packets can only represent energy of photons, but not their electric field. To circumvent this, an MC approach incorporating a new detection scheme derived from the EHF principle was proposed. The approach adopts the 4F optic system as the sample arm geometry and is designed to estimate a heterodyne factor at the q‐plane where a tissue discontinuity is situated. Photon packets are diffusively reflected from the tissue discontinuity and back‐propagated to the p‐plane. Since the p‐plane and q‐plane are unity conjugates, it was proved that the reflected sample beam at the p‐plane is delta‐correlated and the heterodyne efficiency factor calculated in the p‐plane is identical to that calculated in the r‐plane due to the discontinuity in the q‐plane. Consequently, the proposed detection scheme only needs to take into account the intensity distribution of the sample and reference field in the p‐plane. Let Φ_p_ and Φ_r_ be the heterodyne efficiency factor in the p‐plane and the r‐plane, respectively. According to the EHF model, Φ_p_ is expressed as

(15) $$\Phi_{p}=\frac{\left\langle i_{p}^{2}\right\rangle }{\left\langle i_{0,p}^{2}\right\rangle }=\frac{\int I_{R}(p)\langle I_{S}(p)\rangle dp}{\int I_{R}(p)\langle I_{S,0}(p)\rangle dp}=\Phi_{r},$$

where **p** is a point in the p‐plane. I_R_(**p**) is the intensity of the reference beam at **p** in the p‐plane. I_S_(**p**) is the intensity of the back‐propagated sample field through the scattering medium. I_S,0_ is the intensity of the sample field in the absence of the scattering medium. The triangular bracket indicates ensemble averaging over the scattering medium and the diffusively reflecting discontinuity. By modulating the weight of all photon packets propagated to the p‐plane by the intensity distribution of the reference field followed by summation, as shown in Equation 16, the mean squared intensity of the OCT signal due to light reflected from the q‐plane at depth z can be estimated.

(16) $$\langle i^{2}(z)\rangle =\langle i_{p}^{2}\rangle =\Phi_{p}\langle i_{0,p}^{2}\rangle \approx \sum_{p}I_{R}(p)W_{p},$$

where **p** represents a photon packet in the p‐plane and W_**p**_ is its weight. This is a very interesting result since only the intensity distribution represented by photon packets is needed and no assumptions related to field distributions or wave characteristics are considered upon the photon packets. The approach demonstrated a good agreement with the EHF model. However, it is not clear how the heterodyne efficiency factor is estimated if the tissue discontinuity is not coincident with the focal plane, ie, it is not in the q‐plane. Furthermore, in order to obtain a complete A‐scan, the MC simulation must run repeatedly for the Gaussian beam focused at a different depth of the tissue at a time, ie, implementing a dynamic focusing scheme. This renders the approach very time‐consuming. Besides, it cannot be directly applied to our case since we require the focus of the sample arm beam fixed at a certain depth in FDOCT simulations while only one run of the simulation is needed to obtain a complete A‐scan. Therefore, we adapted Tycho's photon detection scheme so that it works for our case that the focus is fixed at a certain depth while a complete A‐scan can still be acquired with a single run of simulations.

Assume the tissue discontinuity is now at the q^′^‐plane, which is at the depth z within the tissue. We can find a plane (p^′^‐plane) at a distance 2f − (d + z/n) to the left of the collimating lens L1 so that the sample field that is reflected from the q^′^‐plane and arrives at the p^′^‐plane is delta‐correlated with the field in the q^′^‐plane, which means that the heterodyne efficiency factor in the p^′^‐plane is identical to that in the r‐plane due to the tissue discontinuity presented in the q^′^‐plane. Since $\left\langle i_{0}^{2}(z)\rangle =\langle i_{0,p^{\prime }}^{2}\right\rangle$, the mean square heterodyne signal in the p^′^‐plane is also identical to that in the r‐plane. A proof of this is provided in Appendix A. Therefore, we are able to estimate the mean squared heterodyne signal by summing up the weight of photon packets modulated by the intensity distribution of the reference field propagated to the p^′^‐plane. As we are interested in the mean square heterodyne signal rather than the heterodyne efficiency factor, we do not need to estimate the heterodyne signal in the absence of the scattering medium. The intensity distribution of the reference field is Gaussian with one parameter $w_{p}^{\prime }$, the 1/e radius of the reference beam in the p^′^‐plane, and it is expressed as

(17) $$I_{R}(p)=\frac{1}{w_{p^{\prime }}^{2}}\exp\left(-\frac{\|p{\|}^{2}}{w_{p^{\prime }}^{2}}\right)$$

In fact, the distance between the p^′^‐plane and the q^′^‐plane should be always 4f. As such, there does exist a limitation that the scanned depth z cannot exceed 2f − d, although it can be circumvented by using a larger f. Here, we summarize our detection scheme in Algorithm 1.

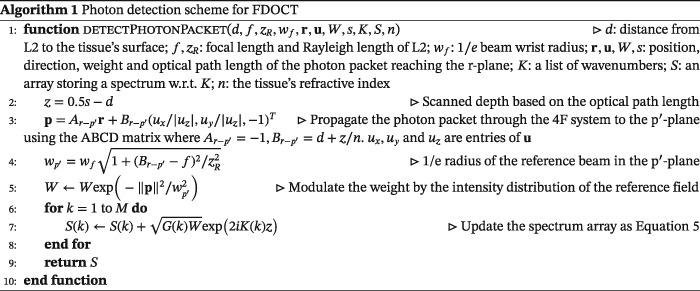

## 4 SIMULATION RESULTS

In this section, we present some simulation results of OCT signals using the MC method described above. To validate the MC method for OCT simulation, A‐scans obtained using the MC approach were compared with the analytical EHF model in the first subsection. In our simulation, we set the absorption coefficient of tissues to zero. Since the EHF model does not include absorption coefficient, we will not be able to compare the simulation results with the EHF model if we allow nonzero absorption coefficients for the simulation. Furthermore, its value is much smaller than the scattering coefficient for tissues in general. In the second subsection, we fit the EHF model to a set of simulation results to see if optical properties can be recovered correctly. We used 10^8^ photon packets in all simulations. All simulations were performed on a desktop computer equipped with an Intel(R) Core i7 CPU at 3.60GHz and 24GB RAM. To demonstrate the performance of the MC approach, we listed the average simulation time for generating an A‐scan for a few types of tissues in Table 1. The ratio of detected photon packets is also summarized. We found that the simulation time is largely dependent on μ_s_ and p_b_, but not g. As μ_s_ and p_b_ increase, the probability for photon packets to be backscattered gets higher leading to more detected photon packets. In the photon detection scheme, the calculation of the intensity of the reference beam involves exponentials, which is time consuming. Therefore, the simulation time is in proportion to the amount of detected photon packets. For all simulations, the light source has a central wavelength of 1300 nm and a bandwidth of 95 nm. The spectrum of wavelength contains 2048 cells and the wavelength spacing is 0.055 nm. The bandwidth of the light source is 229 nm. In order to reduce speckle noises existed in all simulated A‐scans, for each run of MC simulations, a B‐scan along the y‐axis consisting of 10 A‐scans was obtained and the averaged 10‐based logarithm of A‐scans was calculated. For convenience, we refer A‐scan in the following sections as the averaged A‐scan after taking ten‐based logarithm. We converted the spectral data collected from simulations into A‐scans by multiplying the data with a Hamming window followed by the fast Fourier transform. The Hamming window works as a tapering function to reduce the spectral leakage caused by a windowing effect introduced by finite data series. Each A‐scan contains about 300 values at evenly spaced depth within a 1‐mm scan range.

**Table 1:** The average simulation time of one A‐scan in seconds and the ratio of detected photon packets in a percentage of total simulated photon packets (as shown in brackets)

		μ_s_, mm^−1^		
		1	5	10
p_b_	0.01	42 (0.18%)	125 (1.14%)	298 (3.58%)
	0.1	84 (1.65%)	292 (5.98%)	505 (9.13%)

### 4.1 Comparing MC and EHF

**Figure 4:**
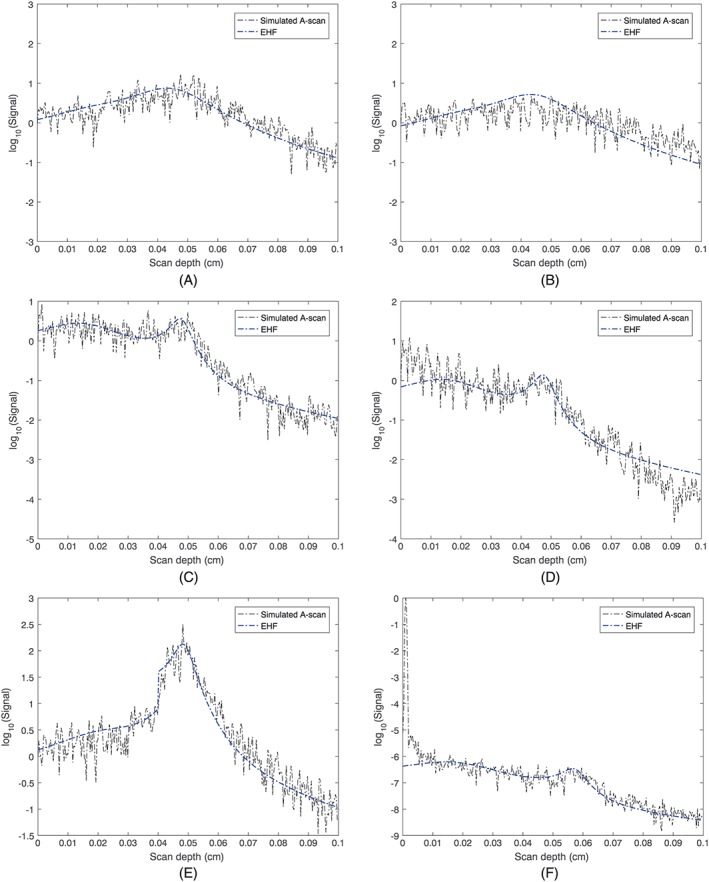
Comparing simulation results of our MC approach with the analytical EHF model. (A, C, E, F) Simulated A‐scans using the proposed photon detection scheme. (B, D) Simulated A‐scans using the original photon detection scheme of MCML. (E) A simulated A‐scan of a two‐layer tissue. The thickness of the first layer is 0.04 cm. (F) A simulated A‐scan of a single layer tissue with a nonunity refractive index n = 1.2. The optical properties of tissues and the radius of the sample beam w_0_ take the following values: (A, B) $\mu_{s}=1\;{\text{mm}}^{-1}$, g = 0.95, p_b_ = 0.05, w_0_ = 0.02 mm; (C, D, F) $\mu_{s}=5\;{\text{mm}}^{-1}$, g = 0.95, p_b_ = 0.05, w_0_ = 0.04 mm. (E): $\mu_{s}=2\;{\text{mm}}^{-1}$, g = 0.95, p_b_ = 0.05 for the first layer; $\mu_{s}=5\;{\text{mm}}^{-1}$, g = 0.98, p_b_ = 0.1 for the second layer, w_0_ = 0.04 mm

As the EHF model is a proven model for OCT, we compare the results from the MC approach with the EHF model. A brief introduction to the EHF model is given in Appendix B. We firstly demonstrate the consistency between the MC approach and the analytical EHF model using some examples as shown in Figure 4. The focal length of the focusing lens was set to 0.5 mm, and it was positioned on the tissue's surface. The 1/*e* radius of the incident light beam at the focusing lens was set to 0.02 and 0.04 mm, respectively. Since we sare only interested in the shape of mean A‐scans as a result of underlying optical properties rather than the actual magnitude, each A‐scan was normalized by its value at *z* = 0. For comparison, we calculated a benchmark mean squared heterodyne signal by plugging the ground truth values of the optical properties into the EHF model and then aligned it to the simulated A‐scan by minimizing the mean squared error between the simulated A‐scan and the benchmark signal. Figure 4A,C shows comparisons between simulated A‐scans and the mean signal obtained with the EHF model. We also plot in Figure 4B,D simulated A‐scans using the same setup of optics and tissues as Figure 4A,C, respectively, but with the original photon detection scheme of the MCML: A photon packet is detected only if it enters the photon‐collecting optical fiber and its incident angle to the fiber is smaller than the acceptance angle of the fiber. The radius of the fiber is set the same as *w*_*f*_, the waist radius of the incident sample beam. The acceptance angle is calculated as *θ*_*max*_ = *atan*(*w*_0_/*f*), the maximal half‐angle of the cone of light exiting F and entering L1. Noticeably, the new detection scheme achieves a better agreement to the EHF model in terms of signal profiles. Figure 4E shows a simulated A‐scan for a two‐layer tissue. The thickness of the first layer is 0.4 mm. A clear boundary between the two layers is indicated by a rapid rise in the simulated A‐scan at depth *z* = 0.4 mm. A good match between the MC simulation and EHF model is also achieved. In order to show the effect of mismatch in refractive indexes between the tissue and the air, we set the tissue's refractive index to 1.2 and plot a simulated A‐scan in Figure 4F. Note that for this simulation, we launched photon packets from the focusing lens. The difference in refractive indexes introduced a huge peak into the simulated A‐scan suggesting that a large amount of photons were reflected at the surface of the tissue. The EHF model, however, cannot model such reflection at the surface of the tissue.

### 4.2 Parametric fitting

We demonstrate a solution to the inverse problem by fitting the EHF model to the MC simulated A‐scans. We then compared the fitted optical parameters to their ground truth values to show the consistency between the two models. The optical geometry for this is set as follows. The focal length of the focusing lens is 0.5 mm and is positioned immediately next to the tissue's surface. The 1/e radius of the sample arm beam at the focusing lens (in the r‐plane) is set to 0.1 mm. We use a single‐layered tissue for all runs of simulations. The tissue has a depth of 1 mm and is infinitely wide. For simplicity, we assume it has a unity refractive index, the same as surrounding air, as we do not want the light reflection on the surface of the tissue affect our simulation results. We specify a set of values for each optical property of the tissue in order to explore different types of tissues. Specifically, *μ*_*s*_ is set to 1 mm^−1^ for tissues of very low optical scattering, 5 mm^−1^ for low optical scattering and 10 *mm* ^−1^ for high optical scattering respectively. *g* is set to 0.9 for tissues of wide‐angle optical scattering, 0.95 for small‐angle scattering, and 0.98 for highly forward scattering tissues respectively. We set *p*_*b*_ to 0.01 for tissues of low diffusive scattering and 0.1 for highly diffusive scattering. The absorptive coefficient *μ*_*a*_ of all tissues is set to zero. We can combine these values of the optical properties to cover a wide range of tissues.

**Figure 5:**
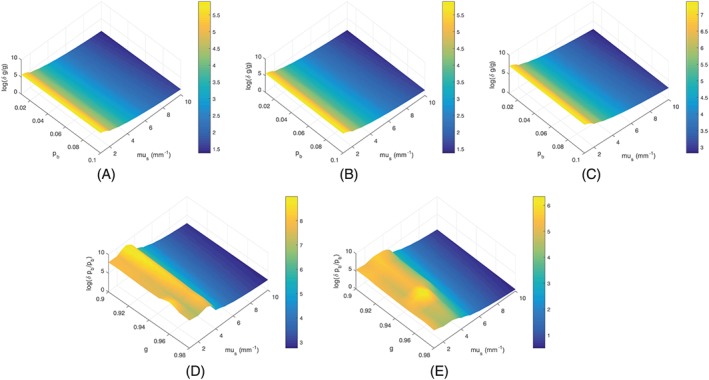
Estimated relative errors of optical properties using linear perturbation analysis on the EHF model. (A) g = 0.9. (B) g = 0.95. (C) g = 0.98. (D) p_b_ = 0.01. (E) p_b_ = 0.1

We used a genetic algorithm for parametric fitting to avoid local optima. We sampled a set of values from the fitted curve at a sequence of depths and used the sum of squared difference between these values sampled from the fitted curve and the simulated A‐scan as minimization criterion for the genetic algorithm. To improve fitting efficiency, we constrain the parameters as follows: 1 ≤ *μ*_*s*_ ≤ 15, 0.8 ≤ *g* ≤ 1, 0.001 ≤ *p*_*b*_ ≤ 0.5, 10^−3^ ≤ *a* ≤ 10^3^, where *a* is a multiplying factor applied to the mean squared heterodyne signal before taking logarithm. We ran MC simulations 10 times on each of the eighteen types of tissues as described earlier. We then calculated the mean and standard deviation of the fitted optical properties for each type of tissues. Estimated values of the optical properties are summarized in Tables 2, 3, and 4. All values are displayed as mean with 95% confidence level. Table 2 shows estimated scattering coefficients *μ*_*s*_ from simulations where *g* and *p*_*b*_ were set to different values. The estimated scattering coefficient is generally in good agreement with their true values. However, there are relatively larger errors in *g* and *p*_*b*_ estimates in the context of small optical scattering and anisotropic factors. It is expected that the MC model and the EHF model are not completely in agreement, since they model light propagation in different ways: One treats photons as particles and propagate them based on the radiative transfer equation and the other is based on theories of wave propagation. Thus, there is always an inherent discrepancy between these two models leading to errors in the inverse problem. To further investigate the cause to the larger errors, we conducted a sensitivity analysis on the EHF model regarding the inverse problem so as to explore how the uncertainty in the measurement (ie, A‐scans) affects estimation of the optical properties. To this end, we derived a measure of the relative error for each optical property from the linear perturbation analysis as follows

(18) $$\frac{\delta u}{u}\leq \frac{1}{N_{z}}\sum_{i=1}^{N_{z}}\left|\frac{\partial u}{\partial I(z_{i})}\frac{I(z_{i})}{u}\right|\left|\frac{\delta I(z_{i})}{I(z_{i})}\right|,$$

where *u* is an optical property to be investigated (ie, *μ*_*s*_, *g* or *p*_*b*_). *I*(*z*) is the A‐scan. *z*_*i*_, *i* = 1,…,*N*_*z*_, are a sequence of depth values sampled within the scan range, which were set identical to the depth values of the simulated A‐scan. *δI*(*z*_*i*_) is the measurement error at depth *z*_*i*_. To obtain the measurement error, we calculated the depth‐resolved standard deviation of the A‐scans for each type of tissues obtained from our MC simulation. We then averaged these standard deviations over all depth values and all types of tissues and used it as the measurement error (2.4 dB). $\frac{\partial u}{\partial I(z_{i})}$ can be calculated directly from the EHF model. In Figure 5A‐C, we plot the upper bound of relative errors of the estimation of *g* for *g* = 0.9, *g* = 0.95, and *g* = 0.98, respectively, with *μ*_*s*_ and *p*_*b*_ set to different values. It can be observed that the relative error of the estimation of *g* drops as *μ*_*s*_ increases, regardless of *p*_*b*_. This implies that in the condition of smaller *μ*_*s*_, it is more difficult to estimate *g* correctly. Figure 5D,E visualizes relative errors of the estimation of *p*_*b*_ for *p*_*b*_ = 0.01 and *p*_*b*_ = 0.1, respectively, when *μ*_*s*_ and *g* vary. Similarly, as *μ*_*s*_ increases, the relative error of the estimation of *p*_*b*_ is reduced. Thus, the sensitivity analysis reveals that in tissues of low scattering, the A‐scan is insensitive to the variation of *g* and *p*_*b*_, which is confirmed by the fitting results presented here.

**Table 2:** Estimated scattering coefficient μ_s_ from parametric fitting using genetic algorithms[Link]

		*g*		
		0.90	0.95	0.98
*p*_*b*_	0.01	1.16 ± 0.40	1.38 ± 1.05	1.34 ± 0.50
		5.13 ± 0.95	5.28 ± 0.75	5.24 ± 0.93
		10.59 ± 1.49	10.64 ± 1.14	10.84 ± 1.99
	0.1	1.15 ± 0.32	1.10 ± 0.36	1.30 ± 0.62
		4.88 ± 0.89	5.12 ± 0.92	5.16 ± 0.63
		10.36 ± 1.28	10.53 ± 1.15	10.90 ± 3.37

^a^Each table cell contains three rows of figures corresponding to ground truth values of *μ*_*s*_: 1, 5, and 10.

**Table 3:** Estimated anisotropy factor g from parametric fitting using genetic algorithms[Link]

		*μ*_*s*_, mm^−1^		
		1	5	10
*p*_*b*_	0.01	0.91 ± 0.13	0.94 ± 0.03	0.94 ± 0.02
		0.96 ± 0.05	0.97 ± 0.02	0.95 ± 0.02
		0.99 ± 0.02	0.99 ± 0.01	0.98 ± 0.01
	0.1	0.90 ± 0.14	0.92 ± 0.06	0.89 ± 0.05
		0.96 ± 0.03	0.96 ± 0.02	0.94 ± 0.02
		0.97 ± 0.08	0.98 ± 0.01	0.97 ± 0.02

^a^Each table cell contains two rows of figures corresponding to ground truth values of *g*: 0.9, 0.95, and 0.98.

**Table 4:** Estimated backscattering probability p_b_ from parametric fitting using genetic algorithms

		*μ*_*s*_, mm^−1^		
		1	5	10
*g*	0.90	0.28 ± 0.38	0.03 ± 0.05	0.001 ± 0
		0.21 ± 0.42	0.10 ± 0.11	0.03 ± 0.02
	0.95	0.20 ± 0.34	0.06 ± 0.09	0.01 ± 0.02
		0.12 ± 0.30	0.14 ± 0.05	0.07 ± 0.02
	0.98	0.14 ± 0.2	0.05 ± 0.04	0.02 ± 0.02
		0.22 ± 0.29	0.15 ± 0.06	0.10 ± 0.02

^a^Each table cell contains three rows of figures corresponding to ground truth values of *p*_*b*_: 0.01 and 0.1.

## 5 CONCLUSION

In this paper, we reviewed main approaches for OCT simulation and presented a new Monte Carlo based approach for simulating FDOCT signals. We consider the incident light beam as a Gaussian light beam, a more realistic assumption than the conventional OCT simulation approaches. Due to the introduction of Gaussian light beams, the conventional photon detection scheme becomes invalid and may cause errors in the simulation result. We addressed this issue by incorporating a photon detection scheme derived from Tycho^22^ into our FDOCT model, which modulates the weight of backscattered photon packets by the intensity profile of the reference light beam. We compared our approach with the analytical EHF model, a popular and proven model for OCT, to demonstrate that the simulated A‐scans using our MC approach are in good agreement with those using the EHF model. By fitting the EHF model to the simulated A‐scans, we demonstrated that the optical properties can be recovered correctly from tissues with high optical scattering coefficients and anisotropy factors. MC however can give more realistic simulation results and deal with more complex tissue structures than EHF. Our future work will involve inverse MC for more robust tissue parameter estimation.

## APPENDIX A PROOF OF EQUIVALENCE OF SQUARED HETERODYNE SIGNALS

### A.1

We prove that the square heterodyne signal in the p^*′*^‐plane, which is at a distance 2*f* − (*d* + *z*/*n*) to the left of L1, is identical to that in the r‐plane resulted from the field reflected from the q^*′*^‐plane at depth *z*. We extend a proof presented in Tycho^22^ for the equivalence of the heterodyne efficiency factor between the p‐plane and the r‐plane. We first prove that the sample field is delta‐correlated in the p^*′*^‐plane with the field in the q^*′*^‐plane in the presence of scattering, ie,

(A1) $$\Gamma_{S}(p_{1}^{\prime },p_{2}^{\prime })=\langle U_{S}(p_{1}^{\prime })U_{S}(p_{2}^{\prime })\rangle \propto \delta (p_{1}^{\prime }-p_{2}^{\prime })\langle I_{S}(p_{1}^{\prime })\rangle ,$$

where $p_{1}^{\prime }$ and $p_{2}^{\prime }$ are vectors in the p^*′*^‐plane. *I*_*S*_(**p**) is the intensity of the sample field. $U_{S}(p^{\prime })$ is the back reflected field by the tissue discontinuity in the q^*′*^‐plane. To find out $U_{S}(p^{\prime })$, we firstly calculate the field at the r‐plane, *U*_*r*_(**r**), by propagating a reflected field $U_{q}(q^{\prime })$ in the q^*′*^‐plane to the r‐plane as

(A2) $$U_{r}(r)=\int U_{q}(q^{\prime })G_{q^{\prime }-r}\exp(i\varphi (q^{\prime },r))dq^{\prime },$$

where ϕ(**q** ^*′*^,**r**) is a random phase of a spherical wave propagating in the scattering medium from the q^*′*^‐plane to the r‐plane. *G*_*q**′* − *r*_ is the Huygens‐Fresnel Green's function of propagating a field from the q^*′*^‐plane to r‐plane given as

(A3) $$G_{q^{\prime }-r}(q^{\prime },r)=-\frac{k}{2\pi B_{q^{\prime }-r}}\exp\left(-\frac{ik}{2B_{q^{\prime }-r}}(A_{q^{\prime }-r}q^{\prime 2}-2q^{\prime }\cdot r+D_{q^{\prime }-r}r^{2})\right).$$

Here, *k* is the wavenumber. *A*_*q**′*  − *r*_  = 1, *B*_*q**′*  − *r*_ = *d* + *z*/*n* and *D*_*q**′*  − *r*_ = 1 are elements of the ABCD matrix of light transfer from the q^*′*^‐plane to the r‐plane. *q* ^*′*^ and *r* are the length of the vector **q** ^*′*^ and **r**, respectively. Secondly, *U*_*r*_(*r*) is propagated to the p^*′*^‐plane through lens L2 and L1, which gives $U_{S}(p^{\prime })$ as

(A4) $$U_{S}(p^{\prime })=\int U_{r}(r)G_{r-p^{\prime }}(r,p^{\prime })dr,$$

where *G*_*r* − *p**′*_ is the Huygens‐Fresnel Green's function for propagating a field from the r‐plane to the p^*′*^‐plane, which has the same form as Equation A3 where the ABCD matrix elements are calculated as

(A5) $$\left(\begin{array}{cc} A_{r-p^{\prime }} & B_{r-p^{\prime }}\\ C_{r-p^{\prime }} & D_{r-p^{\prime }} \end{array}\right)=\left(\begin{array}{cc} 1 & 2f-(d+z/n)\\ 0 & 1 \end{array}\right)\left(\begin{array}{cc} 1 & 0\\ -\frac{1}{f} & 1 \end{array}\right)\left(\begin{array}{cc} 1 & 2f\\ 0 & 1 \end{array}\right)\left(\begin{array}{cc} 1 & 0\\ -\frac{1}{f} & 1 \end{array}\right)=\left(\begin{array}{cc} -1 & d+z/n\\ 0 & -1 \end{array}\right).$$

Therefore, the cross‐correlation of $U_{S}(p^{\prime })$ is calculated as

(A6) $$\begin{array}{ccc} \Gamma_{S}(p_{1}^{\prime },p_{2}^{\prime })=\langle U_{S}(p_{1}^{\prime })U_{S}^{*}(p_{2}^{\prime })\rangle & =⨌G_{q^{\prime }-r}(q_{1}^{\prime },r_{1})G_{q^{\prime }-r}^{*}(q_{2}^{\prime },r_{2})G_{r-p^{\prime }}(r_{1},p_{1}^{\prime })G_{r-p^{\prime }}^{*}(r_{2},p_{2}^{\prime }) & \\ & \;\times \langle U_{q^{\prime }}(q_{1}^{\prime })U_{q^{\prime }}^{*}(q_{2}^{\prime })\rangle \times \langle \exp(i\phi (q_{1}^{\prime },r_{1})-i\phi (q_{2}^{\prime },r_{2}))\rangle dr_{1}dr_{2}dq_{1}^{\prime }dq_{2}^{\prime }. \end{array}$$

Since the sample field is diffusively reflected at the discontinuity, $U_{q^{\prime }}(q^{\prime })$ is delta‐correlated, that is,

(A7) $$\langle U_{q^{\prime }}(q_{1}^{\prime })U_{q^{\prime }}^{*}(q_{2}^{\prime })\rangle =\frac{4\pi }{k^{2}}\delta (q_{1}^{\prime }-q_{2}^{\prime })\langle I_{q^{\prime }}(q^{\prime })\rangle ,$$

where $I_{q^{\prime }}(q^{\prime })$ is the intensity of the field $U_{q^{\prime }}(q^{\prime })$. Thus, Equation A6 becomes

(A8) $$\Gamma_{S}(p_{1}^{\prime },p_{2}^{\prime })\propto ∭G_{q^{\prime }-r}(q^{\prime },r_{1})G_{q^{\prime }-r}^{*}(q^{\prime },r_{2})G_{r-p^{\prime }}(r_{1},p_{1}^{\prime })G_{r-p^{\prime }}^{*}(r_{2},p_{2}^{\prime })\langle I_{q^{\prime }}(q^{\prime })\rangle \Gamma_{pt}(r_{1}-r_{2})dr_{1}dr_{2}dq^{\prime },$$

where $\Gamma_{pt}(r_{1}-r_{2})=\langle \exp(i\phi (q^{\prime },r_{1})-i\phi (q^{\prime },r_{2}))\rangle$, the mutual coherence function of a spherical wave in the r‐plane from a point source in the q^*′*^‐plane. According to the EHF principle, the mean intensity of $U_{q^{\prime }}(q^{\prime })$, $\left\langle I_{q^{\prime }}(q^{\prime })\right\rangle$, is given as

(A9) $$\langle I_{q^{\prime }}(q^{\prime })\rangle ={\left(\frac{k}{2\pi B_{r-q^{\prime }}}\right)}^{2}\int K(\tilde{𝛒})\exp\left(\frac{ik}{B_{r-q^{\prime }}}\tilde{𝛒}\cdot q^{\prime }\right)\Gamma_{pt}(\tilde{𝛒})d\tilde{𝛒},$$

where *B*_*r* − *q*_^*′*^ = *d* + *z*/*n* and $\tilde{𝛒}={\tilde{r}}_{1}-{\tilde{r}}_{2}$. ${\tilde{r}}_{1}$ and ${\tilde{r}}_{2}$ are vectors in the r‐plane. $K(\tilde{𝛒})$ is the overlap integral of the unscattered sample field $U_{S}(\tilde{r})$ in the r‐plane:

(A10) $$K(\tilde{\rho })=\int \exp\left(-\frac{ikA_{r-q^{\prime }}}{B_{r-q^{\prime }}}\tilde{\rho }\cdot \tilde{R}\right)U_{S}(\tilde{R}+\tilde{𝛒}/2)U_{S}^{*}(\tilde{R}-\tilde{𝛒}/2)d\tilde{R},$$

where *A*_*r* − *q*_^*′*^ = 1 and $\tilde{R}=({\tilde{r}}_{1}+{\tilde{r}}_{2})/2$. By integration over **q** ^*′*^ in Equation A8, ie,

(A11) $$\begin{array}{ccc} \int G_{q^{\prime }-r} & \left(q_{1}^{\prime },r_{1})G_{q^{\prime }-r}^{*}(q_{2}^{\prime },r_{2})\langle I_{q^{\prime }}(q^{\prime })\rangle dq^{\prime }={\left(\frac{k}{2\pi (d+z/n)}\right)}^{4}\exp\left(-\frac{ik}{d+z/n}(r_{1}^{2}-r_{2}^{2})\right)K(\tilde{𝛒})\Gamma_{pt}(\tilde{𝛒}\right) & \\ & \times \int \exp\left[\frac{ik}{d+z/n}(q^{\prime }(r_{1}-r_{2}+\tilde{𝛒}))\right]dq^{\prime }\\ & ={\left(\frac{k}{2\pi (d+z/n)}\right)}^{2}\exp\left(-\frac{ik}{d+z/n}(r_{1}^{2}-r_{2}^{2})\right)K(\tilde{𝛒})\Gamma_{pt}(\tilde{𝛒})\delta (r_{1}-r_{2}+\tilde{𝛒}), \end{array}$$

where the last step is due to the expression found in the inverse Fourier transform of the delta function

(A12) $$\int \exp[im\cdot (u+v)]dm={\left(2\pi \right)}^{2}\delta (u+v)$$

and also considering that

(A13) $$G_{r-p^{\prime }}(r_{1},p_{1}^{\prime })G_{r-p^{\prime }}^{*}(r_{2},p_{2}^{\prime })={\left(\frac{k}{2\pi (d+z/n)}\right)}^{2}\exp\left(\frac{ik}{d+z/n}(r_{1}^{2}-r_{2}^{2})\right)\exp\left(\frac{ik}{2(d+z/n)}(2r_{1}p_{1}^{\prime }-2r_{2}p_{2}^{\prime }+p_{1}^{\prime 2}-p_{1}^{\prime 2})\right)$$

Equation A8 becomes

(A14) $$\begin{array}{ccc} \Gamma_{S}(p_{1}^{\prime },p_{2}^{\prime }) & \propto \iint \left\{\int {\left(\frac{k}{2\pi (d+z/n)}\right)}^{4}K(\tilde{𝛒})\Gamma_{pt}(\tilde{𝛒})\delta (r_{1}-r_{2}+\tilde{𝛒})d\tilde{𝛒}\right\} & \\ & \;\times \exp\left(\frac{ik}{2(d+z/n)}(2r_{1}p_{1}^{\prime }-2r_{2}p_{2}^{\prime }+p_{1}^{\prime 2}-p_{2}^{\prime 2})\right)\Gamma_{pt}(r_{1}-r_{2})dr_{1}dr_{2}\\ & =\int {\left(\frac{k}{2\pi (d+z/n)}\right)}^{2}\left\{\int {\left(\frac{k}{2\pi (d+z/n)}\right)}^{2}\exp\left(\frac{ik}{d+z/n}R(p_{1}^{\prime }-p_{2}^{\prime })\right)dR\right\}\\ & \;\times \exp\left(\frac{ik}{2(d+z/n)}[p_{1}^{\prime 2}-p_{2}^{\prime 2}+𝛒(p_{1}^{\prime }+p_{2}^{\prime })]\right)|\Gamma_{pt}(𝛒){|}^{2}K(-𝛒)d𝛒, \end{array}$$

where ***ρ***=**r**_1_ − **r**_2_, and **R** = (**r**_1_ + **r**_2_)/2. Applying Equation A12, we get

(A15) $$\Gamma_{S}(p_{1}^{\prime },p_{2}^{\prime })\propto \delta (p_{1}^{\prime }-p_{2}^{\prime }){\left(\frac{k}{2\pi (d+z/n)}\right)}^{2}\int \exp\left(\frac{ik}{2(d+z/n)}[p_{1}^{\prime 2}-p_{2}^{\prime 2}+𝛒(p_{1}^{\prime }+p_{2}^{\prime })]\right)|\Gamma_{pt}(𝛒){|}^{2}K(-𝛒)d𝛒.$$

$\Gamma_{S}(p_{1}^{\prime },p_{2}^{\prime })$ is nonzero if and only if $p_{1}^{\prime }=p_{2}^{\prime }$. Therefore, Equation A15 is equivalent to

(A16) $$\Gamma_{S}(p_{1}^{\prime },p_{2}^{\prime })\propto \delta (p_{1}^{\prime }-p_{2}^{\prime }){\left(\frac{k}{2\pi (d+z/n)}\right)}^{2}\int \exp\left(\frac{ik}{d+z/n}𝛒\cdot p_{1}^{\prime }\right)|\Gamma_{pt}(𝛒){|}^{2}K(-𝛒)d𝛒=\delta (p_{1}^{\prime }-p_{2}^{\prime })\langle I_{S}(p_{1}^{\prime })\rangle .$$

Therefore, the sample field is delta‐correlated in the p^*′*^‐plane.

Secondly, we show that the heterodyne efficiency factor in the p^*′*^‐plane is identical to that in the r‐plane, ie, Φ_*p*_^*′*^ = Φ_*r*_. From Equation 15, we have

(A17) $$\langle i_{p^{\prime }}^{2}\rangle =\int I_{R}(p^{\prime })\langle I_{S}(p^{\prime })\rangle dp^{\prime }\propto {\left(\frac{k}{2\pi (d+z/n)}\right)}^{2}\iint \exp\left(\frac{ik}{d+z/n}𝛒\cdot p^{\prime }\right)|\Gamma_{pt}(𝛒){|}^{2}K(-𝛒)I_{R}(p^{\prime })d𝛒dp^{\prime }.$$

$I_{R}(p^{\prime })$ is the intensity of the reference field propagating from the r‐plane to the p^*′*^‐plane:

(A18) $$I_{R}(p^{\prime })={\left(\frac{k}{2\pi B_{r-p^{\prime }}}\right)}^{2}\int K_{R}(\tilde{𝛒})\exp\left(\frac{ik}{B_{r-p^{\prime }}}\tilde{𝛒}\cdot p^{\prime }\right)\Gamma_{pt}(\tilde{𝛒})d\tilde{𝛒},$$

where *B*_*r* − *p*_^*′*^ = *d* + *z*/*n* and $\Gamma_{pt}(\tilde{𝛒})=1$ since the reference field is unscattered. $K_{R}(\tilde{𝛒})$ is the overlap integral of the unscattered reference field $U_{R}(\tilde{r})$ in the r‐plane given as

(A19) $$K_{R}(\tilde{\rho })=\int \exp\left(-\frac{ikA_{r-p^{\prime }}}{B_{r-p^{\prime }}}\tilde{\rho }\cdot \tilde{R}\right)U_{R}(\tilde{R}+\tilde{𝛒}/2)U_{R}^{*}(\tilde{R}-\tilde{𝛒}/2)d\tilde{R},$$

where *A*_*r* − *p**′*_ = −1. Considering $U_{R}({\tilde{r}}_{1})U_{R}^{*}({\tilde{r}}_{2})=U_{S}({\tilde{r}}_{1})U_{S}^{*}({\tilde{r}}_{2})$, we have $K_{R}(-\tilde{\rho })=K(\tilde{\rho })$. By plugging Equation A18 into Equation A17, it becomes

(A20) $$\begin{array}{ccc} \left\langle i_{p^{\prime }}^{2}\right\rangle & =\iint \left\{\int {\left(\frac{k}{2\pi (d+z/n)}\right)}^{2}\exp\left(\frac{ik}{d+z/n}(𝛒+\tilde{𝛒})\cdot p^{\prime }\right)dp^{\prime }\right\}{\left(\frac{k}{2\pi (d+z/n)}\right)}^{2}K_{R}(\tilde{𝛒})|\Gamma_{pt}(𝛒){|}^{2}K(-𝛒)d\tilde{𝛒}d𝛒 & \\ & =\int {\left(\frac{k}{2\pi (d+z/n)}\right)}^{2}\left\{\int \delta (𝛒+\tilde{𝛒})K_{R}(\tilde{𝛒})d\tilde{𝛒}\right\}|\Gamma_{pt}(𝛒){|}^{2}K(-𝛒)d𝛒={\left(\frac{k}{2\pi (d+z/n)}\right)}^{2}\int |\Gamma_{pt}(𝛒){|}^{2}|K(𝛒){|}^{2}d𝛒. \end{array}$$

$\left\langle i_{0,p^{\prime }}^{2}\right\rangle$ is obtained by simply setting Γ_*pt*_(***ρ***) in the last step of Equation A20 to unity in the absence of scattering media, that is,

(A21) $$\langle i_{0,p^{\prime }}^{2}\rangle ={\left(\frac{k}{2\pi (d+z/n)}\right)}^{2}\int |K(𝛒){|}^{2}d𝛒.$$

Therefore, the heterodyne efficiency factor is calculated as

(A22) $$\Phi_{p^{\prime }}=\frac{\left\langle i_{p^{\prime }}^{2}\right\rangle }{\left\langle i_{0,p^{\prime }}^{2}\right\rangle }=\frac{\int |\Gamma_{pt}(𝛒){|}^{2}|K(𝛒){|}^{2}d𝛒}{\int |K(𝛒){|}^{2}d𝛒}.$$

Thirdly, we show that

(A23) $$\Phi_{r}=\frac{\int |\Gamma_{pt}(𝛒){|}^{2}|K(𝛒){|}^{2}d𝛒}{\int |K(𝛒){|}^{2}d𝛒}.$$

The mean square of heterodyne signal measured in the r‐plane due to light back‐scattered from the q^*′*^‐plane at depth *z* is given by^3^

(A24) $$\langle i^{2}(z)\rangle \propto \iint \Gamma_{S}(r_{1},r_{2};z)\Gamma_{R}(r_{1},r_{2})dr_{1}dr_{2},$$

where $\Gamma_{R}(r_{1},r_{2})=U_{R}(r_{1})U_{R}^{*}(r_{2})$, and

(A25) $$\begin{array}{ccc} \Gamma_{S}(r_{1},r_{2};z) & =\iint \langle U_{q^{\prime }}(q_{1}^{\prime })U_{q^{\prime }}^{*}(q_{2}^{\prime }){\tilde{G}}_{q^{\prime }-r}(q_{1}^{\prime },r_{1}){\tilde{G}}_{q^{\prime }-r}^{*}(q_{2}^{\prime },r_{2})\rangle dr_{1}dr_{2} & \\ & =\iint \langle U_{q^{\prime }}(q_{1}^{\prime })U_{q^{\prime }}^{*}(q_{2}^{\prime })\rangle \langle {\tilde{G}}_{q^{\prime }-r}(q_{1}^{\prime },r_{1}){\tilde{G}}_{q^{\prime }-r}^{*}(q_{2}^{\prime },r_{2})\rangle dr_{1}dr_{2}, \end{array}$$

where ${\tilde{G}}_{q^{\prime }-r}(q^{\prime },r)=G_{q^{\prime }-r}(q^{\prime },r)\exp(i\phi (q^{\prime },r))$. By plugging Equation A7 into Equation A25, it becomes

(A26) $$\begin{array}{ccc} \Gamma_{S}( & r_{1},r_{2};z)\propto \int \langle I_{q^{\prime }}(q^{\prime })\rangle G_{q^{\prime }-r}(q^{\prime },r_{1})G_{q^{\prime }-r}^{*}(q^{\prime },r_{2})\Gamma_{pt}(r_{1}-r_{2})dq^{\prime } & \\ & ={\left(\frac{k}{2\pi B_{q^{\prime }-r}}\right)}^{2}\int \left\{\int {\left(\frac{k}{2\pi B_{r-q^{\prime }}}\right)}^{2}K(\tilde{𝛒})\exp\left(\frac{ik\tilde{𝛒}\cdot q^{\prime }}{B_{r-q^{\prime }}}\right)\Gamma_{pt}(\tilde{𝛒})d\tilde{𝛒}\right\}\exp\left[\frac{ik}{B_{q^{\prime }-r}}(𝛒\cdot q^{\prime }-𝛒\cdot R)\right]\Gamma_{pt}(𝛒)dq^{\prime }\\ & ={\left(\frac{k}{2\pi (d+z/n)}\right)}^{2}\int \left\{\int {\left(\frac{k}{2\pi (d+z/n)}\right)}^{2}\exp\left(\frac{ik(𝛒+\tilde{𝛒})\cdot q^{\prime }}{d+z/n}\right)dq^{\prime }\right\}K(\tilde{𝛒})\Gamma_{pt}(\tilde{𝛒})\Gamma_{pt}(𝛒)\exp\left(-\frac{ik}{d+z/n}𝛒\cdot R\right)d\tilde{𝛒}. \end{array}$$

The last step is due to **B**_*q**′* − *r*_ = **B**_*r* − *q*′_ = *d* + *z*/*n*. Using Equation (A12), the equation above is simplified to

(A27) $$\Gamma_{S}(r_{1},r_{2};z)\propto {\left(\frac{k}{2\pi (d+z/n)}\right)}^{2}K(-𝛒)|\Gamma_{pt}(𝛒){|}^{2}\exp\left(-\frac{ik}{d+z/n}𝛒\cdot R\right).$$

By plugging Equation A27 into Equation A24 and assuming that $U_{R}(r_{1})U_{R}^{*}(r_{2})=U_{S}(r_{1})U_{S}^{*}(r_{2})$, the mean square of heterodyne signal in the r‐plane is given as

(A28) $$\begin{array}{ccc} \left\langle i^{2}(z)\right\rangle & \propto {\left(\frac{k}{2\pi (d+z/n)}\right)}^{2}\int d𝛒\int \exp\left(-\frac{ik}{d+z/n}𝛒\cdot R\right)U_{S}(R+𝛒/2)U_{S}(R-𝛒/2)dR\times & \\ & K(-𝛒)|\Gamma_{pt}(𝛒){|}^{2}={\left(\frac{k}{2\pi (d+z/n)}\right)}^{2}\int |K(𝛒){|}^{2}|\Gamma_{pt}(𝛒){|}^{2}d𝛒. \end{array}$$

By setting Γ_*pt*_(***ρ***) to unity, the mean square of heterodyne signal in the absence of the scattering medium is obtained. The heterodyne efficiency factor in the r‐plane is calculated as

(A29) $$\Phi_{r}=\frac{\left\langle i^{2}(z)\right\rangle }{\left\langle i_{0}^{2}(z)\right\rangle }=\frac{\int |\Gamma_{pt}(𝛒){|}^{2}|K(𝛒){|}^{2}d𝛒}{\int |K(𝛒){|}^{2}d𝛒}=\Phi_{p^{\prime }}.$$

Since $\left\langle i_{0}^{2}(z)\rangle =\langle i_{0,p^{\prime }}^{2}\right\rangle$, the mean square heterodyne signal in the p^*′*^‐plane is also identical to that in the r‐plane.

## APPENDIX B THE EHF MODEL OF OCT SIGNALS

### B.1

Let *μ*_*s*_, *g*, and *p*_*b*_ be the scattering coefficient, the anisotropy factor, and the backscattering probability of a tissue. The tissue has a refractive index *n*. For the settings of the OCT system, the sample arm beam with a radius *w*_0_ is focused into the tissue by a focusing lens with a focus length *f*. The distance from the lens to the tissue surface is *d*. Let *k* be the wavenumber of the probing light beam: *k* = 2*π*/*λ*, where *λ* is the wavelength. The mean square heterodyne signal current *i*(*z*) at depth *z* can be formulated as

(B1) $$\langle i^{2}(z)\rangle ={\left\langle i^{2}(z)\right\rangle }_{0}\Psi (z),$$

where ⟨*i* ^2^(*z*)⟩_0_ is the mean square heterodyne signal current in the absence of scattering at depth *z*, Ψ(*z*) is the heterodyne efficiency factor representing signal degradation due to scattering. ⟨*i* ^2^(*z*)⟩_0_ is defined as

(B2) $${\left\langle i^{2}(z)\right\rangle }_{0}=\frac{\alpha^{2}P_{R}P_{S}\mu_{s}p_{b}}{\pi w_{H}^{2}(z)},$$

where *α* is the power to current conversion factor, *P*_*R*_ and *P*_*S*_ is the power of the reference and the sample arm beams, and *w*_*H*_(*z*) is the 1/*e* radius of the irradiance at the probing depth *z* defined as

(B3) $$w_{H}^{2}(z)=w_{0}^{2}{\left(A-\frac{B(z)}{f}\right)}^{2}+{\left(\frac{B(z)}{kw_{0}}\right)}^{2}.$$

*A* and *B*(*z*) are entries of the ABCD ray‐matrix for light propagation from the lens plane to the probing depth. For this case where the sample arm light beam is focused on the surface of the tissue, we have *A* = 1 and *B*(*z*) = *d* + *z*/*n*.

(B4) $$\Psi (z)=\exp(-2\mu_{s}z)+\frac{4\exp(-\mu_{s}z-p_{b}\mu_{s}z)[1-\exp(-\mu_{s}z)]}{1+w_{S}^{2}(z)/w_{H}^{2}(z)}+{\left[1-\exp(-\mu_{s}z)\right]}^{2}\exp(-2p_{b}\mu_{s}z)\frac{w_{H}^{2}(z)}{w_{S}^{2}(z)}.$$

Here, $w_{S}^{2}(z)$ is the 1/*e* irradiance radius of the light beam at depth *z* with presented scattering, which is calculated as

(B5) $$w_{S}^{2}(z)=w_{H}^{2}(z)+{\left(\frac{2B(z)}{k\rho_{0}(z)}\right)}^{2}.$$

*ρ*_0_(*z*) is the lateral coherence length defined as follows

(B6) $$\rho_{0}(z)=\sqrt{\frac{3}{\mu_{s}z}}\frac{\lambda }{\pi \theta_{rms}\sqrt{1-2p_{b}}}\left(\frac{nB(z)}{z}\right)=\sqrt{\frac{3}{\mu_{s}z}}\frac{\lambda }{\pi \theta_{rms}\sqrt{1-2p_{b}}}\left(1+\frac{nd}{z}\right),$$

where *θ*_*rms*_, the root‐mean‐square of the scattering angle, is calculated as $\theta_{rms}=\sqrt{2(1-g)}$. Equation B4 contains three items: The first item represents the component of the probing light undergoing single scattering; the third item is the component experiencing multiple scattering due to tissue inhomogeneity, and the second item is the coherence mixing of the unscattered and multiple scattered light.
